# Phytochemical Analysis and Habitat Suitability Mapping of *Glycyrrhiza glabra* L. Collected in the Hatay Region of Turkey

**DOI:** 10.3390/molecules25235529

**Published:** 2020-11-25

**Authors:** Doaa H. M. Alsaadi, Aedla Raju, Ken Kusakari, Faruk Karahan, Nazim Sekeroglu, Takashi Watanabe

**Affiliations:** 1 Department of Medicinal Plant, Graduate School of Pharmaceutical Sciences, Kumamoto University, 5-1 Oe-Honmachi, Chuo-ku, Kumamoto, Kumamoto 862-0973, Japan; 182y2051@st.kumamoto-u.ac.jp (D.H.M.A.); kusaken@kumamoto-u.ac.jp (K.K.); wtakashi@kumamoto-u.ac.jp (T.W.); 2 Global Center for Natural Resources Sciences, Kumamoto University, No. 5-1, Oe Honmachi, Chuo-ku, Kumamoto 862-0973, Japan; 3 Department of Biology, Faculty of Science and Literature, Hatay Mustafa Kemal University, 31060 Hatay, Turkey; fkarahan@mku.edu.tr; 4 Department of Horticulture, Faculty of Agricultural Engineering, Kilis 7 Aralik University, 79000 Kilis, Turkey; sekeroglu@kilis.edu.tr; 5 Advanced Technology Application and Research Center (ATARC), Kilis 7 Aralik University, 79000 Kilis, Turkey

**Keywords:** frequency ratio, glabridin, glycyrrhizic acid, habitat suitability map, liquiritin, soil moisture content

## Abstract

The growth and quality of licorice depend on various environmental factors, including the local climate and soil properties; therefore, its cultivation is often unsuccessful. The current study investigated the key factors that affect the contents of bioactive compounds of *Glycyrrhiza glabra* L. root and estimated suitable growth zones from collection sites in the Hatay region of Turkey. The contents of three bioactive compounds (glycyrrhizic acid, glabridin, and liquiritin), soil factors (pH, soil bearing capacity, and moisture content), and geographical information (slope, aspect, curvature, elevation, and hillshade) were measured. Meteorological data (temperature and precipitation) were also obtained. An analysis of variance (ANOVA) and multivariate analysis of variance (MANOVA) were performed on the data. The soil bearing capacity, moisture content, slope, aspect, curvature, and elevation of the study area showed statistically significant effects on the glycyrrhizic acid and liquiritin contents. A habitat suitability zone map was generated using a GIS-based frequency ratio (FR) model with spatial correlations to the soil, topographical, and meteorological data. The final map categorized the study area into four zones: very high (15.14%), high (31.50%), moderate (40.25%), and low suitability (13.11%). High suitability zones are recommended for further investigation and future cultivation of *G. glabra*.

## 1. Introduction

*Glycyrrhiza*, also known as licorice, is a perennial herbal plant belonging to the Fabaceae family. There are approximately 20 known species of *Glycyrrhiza* distributed worldwide that have great economic value [1]. *Glycyrrhiza* has wide applications in traditional medicines, cosmetics, and the food industry owing to its pharmacological properties and sweet taste. Glycyrrhizic acid, glabridin, liquiritin, isoliquiritin, licoflavanone, pinocembrin, and prunetin are some of many bioactive compounds that can be isolated from licorice root and leaves, and are responsible for its wide range of activities, including anti-inflammatory, antioxidant, and anti-proliferative effects [2,3].

However, due to the wide distribution of licorice, the bioactive compound contents and their biological activities can vary widely both in and between countries depending on geographical and environmental factors [4,5]. In South Korea and China, the quality of licorice root is defined by its glycyrrhizic acid and liquiritin contents according to the pharmacopeia of these countries. Similarly, *G. glabra* and *G. uralensis* are listed in the Japanese pharmacopoeia, which requires a glycyrrhizic acid content of at least 2.0% (dry weight) to ensure quality [6,7]. Despite the efforts to cultivate high quality *G. glabra* in Japan, the obtained product often fails to satisfy this standard [8]; hence, Japanese companies sell imported licorice from China, Europe, and the Middle East [9,10].

Studies have revealed that environmental factors and soil conditions are important for plant growth and the contents of bioactive compounds [11,12]. For instance, soil moisture can affect root growth by causing the accumulation of heavy metals and/or minerals to toxic concentrations [13,14]. Moreover, a high moisture content is usually associated with a low oxygen content (low aeration), low drainage, high redox potential, and high organic matter content. Such conditions also favor the accumulation of magnesium and/or sulfides to a toxic level, which negatively affects plants [15,16].

Geographic information systems (GIS) have been employed to make these parameters more understandable and easier to track. Utilizing GIS-based methods can be beneficial for determining the best growth environment by exploring the resources of *G. glabra* in countries where it grows naturally, and for identifying factors that control its growth [17].

*Glycyrrhiza glabra* grows naturally in the Hatay region of Turkey, which was selected as the study area for this research. This region is characterized by a Mediterranean climate and has adequate amounts of micronutrients in the soil, thus making it highly suitable for the growth of *G. glabra* and other Mediterranean plants [4,18]. The present study investigated the soil, topographical, and environmental characteristics of this area as well as the contents of three bioactive compounds in *G. glabra* (glycyrrhizic acid, glabridin, and liquiritin) in order to highlight the parameters that influence the bioactive contents and distribution of *G. glabra*. The obtained results will improve future investigations and cultivations of *G. glabra* in the Hatay region and other closely related areas.

## 2. Results and Discussion

### 2.1. Chemical Content Analysis and Statistical Analysis

**Figure 1:**
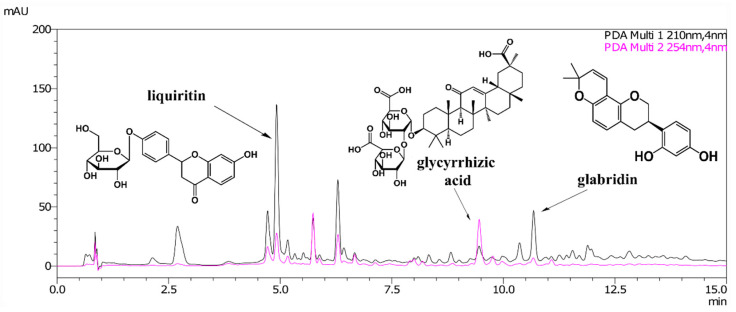
High-performance liquid chromatography (HPLC) chromatogram for the 50% ethanol extract of *G. glabra* roots.

A typical high-performance liquid chromatography (HPLC) chromatogram is shown in Figure 1. Glabridin and liquiritin were detected efficiently at UV 210 nm, whereas glycyrrhizic acid was detected at 254 nm.

The content varied greatly, even at the same location. The glycyrrhizic acid, glabridin, and liquiritin contents ranged from 0.54% to 2.40%, 0.02% to 0.31%, and 0.18% to 1.85%, respectively. Only three samples (E-1, F-2, and F-3) met the Japanese Pharmacopoeia specification (glycyrrhizic acid content of ≥ 2%) (Table 1). Large variations in these compounds have also been reported in previous studies, which were mainly attributed to differences in genetics [19,20] as well as environmental and/or soil parameters [4]. From the statistical results, the plant samples exhibited a weak correlation between the glycyrrhizic acid and liquiritin contents (r = 0.59, *p* < 0.001).

The soils in the study area were neutral to slightly acidic in nature and dry to slightly wet, with a volumetric soil moisture content (VSMC) ranging from 0.12 to 0.33. The soils had a soil bearing capacity of between 2.61 t sf^−1^ to 4.50 t sf^−1^ with different percentages of clay, loam, silt, and sand. These characteristics make the soils less susceptible to disruption, fissuring, and submersion [21]. *Glycyrrhiza glabra* appears to adopt these soil conditions.

The VSMC values increased with increasing altitude (r = 0.92, *p* < 0.001) and increasing slope degree (r = 0.82, *p* < 0.001), which was related to a high level of rainfall. In addition, the MANOVA (using Wilks’ test) and ANOVA analyses revealed that the glycyrrhizic acid and liquiritin contents of *G. glabra* were significantly affected by the soil bearing capacity, VSMC, slope, aspect, curvature, and elevation of the study area. However, the main effective parameters still to be defined. In contrast, the glabridin content was not correlated with the tested variables. These results are similar to those of Esmaeili et al. [20], who suggested that the glabridin content of *G. glabra* was affected by genetic diversity. Furthermore, the meteorological variables and soil pH exhibited no effect with the bioactive contents (Table 2).

The limited size of the study area could explain the minor influence of climate on the contents of bioactive compounds. Moreover, the low variation in the soil pH value may be attributable to the ability of nitrate and other micronutrients to maintain the pH within a certain range. High levels of potassium, calcium, and magnesium have been reported from the soil of the Hatay region [4]. Our soil analysis on five points within the study area also produced similar results (Supplementary Materials Table S4).

### 2.2. Habitat Suitability Zone Map

**Figure 2:**
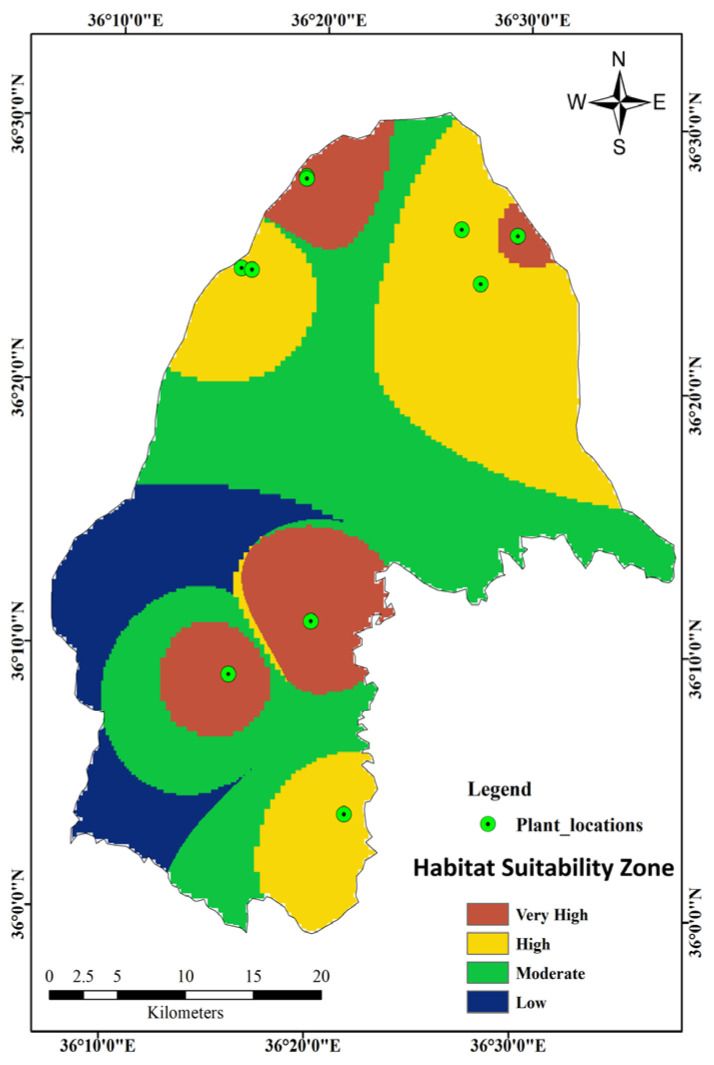
Habitat suitability zone map of *G. glabra* based on the frequency ratio (FR) model.

The final map is presented in Figure 2, and reveals that plant locations C, D, F, H, and J were in very high suitability zones, whereas A, B, E, G, and I were in high suitability zones based on the FR model. The very high suitability zones had a slope of 0–15° facing the south, southwest, and west. The mountain regions, developed areas, and agricultural sites were all within the moderate and low suitability zones, whereas no plant locations were within these zones. From the habitat suitability zone map (HSZM), 15.14%, 31.50%, 40.25%, and 13.11% of the total study area were of a very high suitability, high suitability, moderate suitability, and low suitability, respectively, for *G. glabra* cultivation and future field surveys.

From the curvature map in Figure 5C, the very high suitability of locations C and D were situated in flat areas, while F and J were located at foothills with richer soil and H was in a concave–convex region. Therefore, the possibility of storing rainfall in convex regions (runoff from concave areas) is high. For the high suitability, locations A, B, E, and G were situated in flat areas. Location I was located in a complex concave–flat–convex area, which was at the foothills. As a result, rainfall accumulation was enough for plant growth (Figure 5J). Very high and high suitability zones have soil pH ranged from 6.5 to 6.6 and 6.8 to 7.0. Locations with a low soil bearing capacity (<4.0 mm) and low soil moisture content (<0.24) were considered to be highly suitable zones (Figure 5G,H).

Overall, it was observed that most of the northern region is cultivation land, whereas the central region is urban, and the southern region is mountainous with geographical structures. Location I and J were near the roads. However, the effect of fuel combustion pollutants on the chemical composition was neglected since these roads were mostly not busy with traffic. The mean annual temperature and precipitation were within a range that could encourage agriculture activities and plant growth. The geography and environmental conditions of the study area are appropriate for the extensive distribution of *G. glabra*, as confirmed by field investigations. Moreover, samples from high suitability zones contained relatively good levels of bioactive compounds, specifically F-3, which had the highest glycyrrhizic acid and liquiritin contents.

### 2.3. Validation of the Habitat Suitability Map of G. glabra

**Figure 3:**
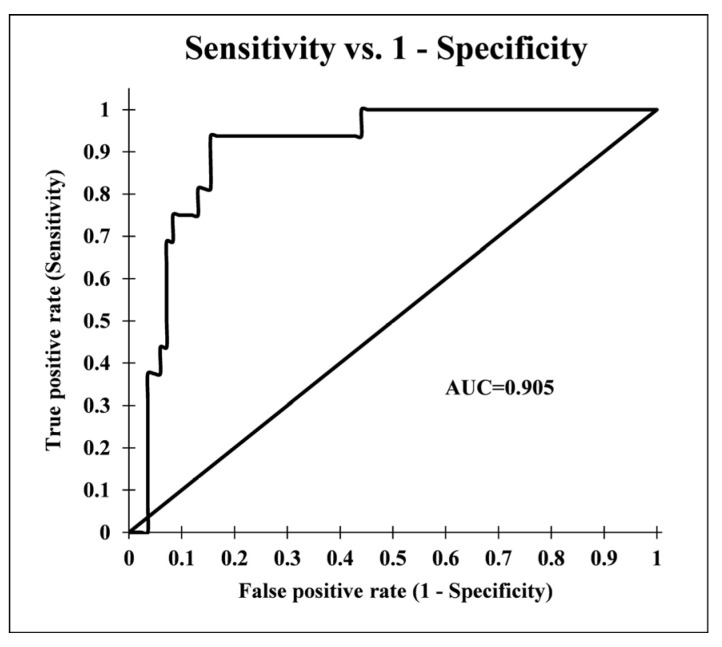
Receiver operator characteristic (ROC) curve for the habitat suitability map of *G. glabra* produced by the frequency ratio model.

The results showed that the FR model had an excellent performance with an AUC value of 0.905 (Figure 3). It can be concluded that the FR model used in this study produced a logical and satisfactory output with a good accuracy for predicting the habitat suitability of *G. glabra* in the study area.

## 3. Materials and Methods

### 3.1. Study Area

**Figure 4:**
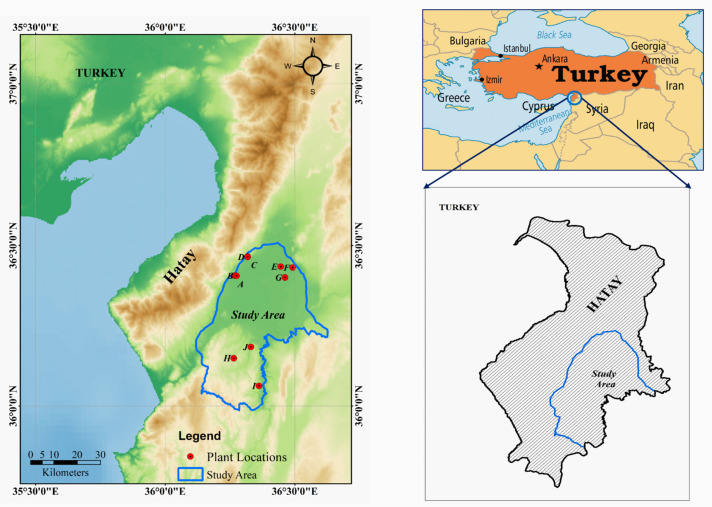
Location map of the study area, which covers an area of 1354 km^2^ in the Hatay region of Turkey. The map shows the 10 sampling locations (red dots), and was created using Google Earth Pro and ArcGIS version 10.5.1. (Licensed).

As mentioned, the study area is situated in the Hatay region of Turkey (36°0′00″–36°35′00″ E, 36°0′00″–36°30′00″ N) and covers an area of 1354 km^2^ (as pinpointed by Google Earth Pro and ArcGIS 10.5.1 version (ESRI Japan Corporation, Tokyo, Japan)) (Figure 4). The northern and central regions of the study area are flat and covered with urban areas and agricultural fields. The southern region is characterized by a mountainous landscape and geological variations. According to the digital elevation model (DEM), the elevation ranges from 75 m to 328 m above sea level, and the slope angle varies from 0° to 52°.

### 3.2. Plant Materials

In November 2019, a total of 28 samples of wild G. glabra roots (diameter of 6–27 mm) were collected from 10 locations (A–J) within the Hatay region (three samples per location except for location A, which had one sample). A global positioning system (GPS) (Garmin eTrex 30x, Olathe, KS, USA) was used to indicate the sampling sites. In-situ soil analysis was performed. The recorded annual average temperature and precipitation (2019) as well as the climate classification for each site were acquired from the Turkish Ministry of Agriculture and Forestry, General Directorate of Meteorology (2020) [22], as shown in Table 3. The plant samples were dried for 7 d at 50 °C, and each sample was then blended into a powder for the extraction experiment.

### 3.3. Chemicals

The COSMOSIL Protein-R column was purchased from Nacalai Tesque (Kyoto, Japan). Dacapo DX-C18 was obtained from Imtakt (Kyoto, Japan). Glycyrrhizic acid, glabridin, and other chemicals were purchased from FUJIFILM Wako Pure Chemical Industries (Osaka, Japan).

### 3.4. Instrumentation

A prominence HPLC instrument (Shimadzu, Kyoto, Japan) was used for chromatographic purification. A Nexera X2 HPLC/UHPLC system (Shimadzu, Japan) was employed for the quantitative analysis. An amaZon speed-ion trap mass spectrometer (Bruker, Billerica, MA, USA) and an AVANCE-I 600 NMR (Bruker) were used for chemical identification. A Yamanaka-type soil hardness tester from Fujiwara Seisakusho, Ltd. (Tokyo, Japan) and soil moisture sensor kit SM150T from Delta-T Devices (Cambridge, UK) were used for the in-situ soil analysis.

### 3.5. Isolation and Identification of Liquiritin

A sample of licorice root was purchased from Uchida Wakanyaku Ltd. (Tokyo, Japan) and ground into a fine powder. The powdered root (100 g) was extracted with 50% ethanol (1 L twice) to obtain approximately 15 g of dry extract. The extract was suspended in water and extracted three times with ethyl acetate, which yielded approximately 2.5 g of the ethyl acetate fraction. The fraction (100 mg mL^−1^ in methanol) was purified by preparative HPLC using a COSMOSIL Protein-R column (20 mm × 250 mm, 5 µm) under the following conditions: elution with methanol–water linear gradient (40:60 at 0 min to 76:24 at 30 min), 8 mL min^−1^ flow rate, 40 °C column temperature, 1 mL injection, and detection by a photodiode array (PDA) detector (200−360 nm). A peak observed between 14.1 min and 15.3 min was collected repeatedly to obtain approximately 10 mg of the isolated compound.

The following matched the values of liquiritin cited in relevant literature [24,25]: slightly yellow powder, ESI-MS (ion-trap) *m*/*z*: [M–H]^−^ 417.3; ^1^H NMR (600 MHz, DMSO-*d*_6_): δ 7.64 (1H, d, *J* = 8.64 Hz, 5-H), 7.45 (2H, d, *J* = 8.76 Hz, 2′-H, 6′-H) 7.07 (2H, d, *J* = 8.76 Hz, 3′-H, 5′-H), 6.51 (1H, dd, *J* = 8.64, 2.22 Hz, 6-H), 6.35 (1H, d, *J* = 2.22 Hz, 8-H), 5.52 (1H, dd, *J* = 12.72, 2.82 Hz, 2-H), 4.89 (1H, d, *J* = 7.44 Hz, 1″-H), 3.7 (1H, d, *J* = 11.16 Hz, 6″-H*α*), 3.46 (1H, m, *J* = 5.7 Hz, 6″-H*β*), 3.13 (1H, m, *J* = 4.95 Hz, 3-H*α*), 2.67 (1H, m, *J* = 4.4 Hz, 3-H*β*); ^13^C NMR (150 MHz, DMSO-*d*_6_): δ 78.23 (C-2), 44.98 (C-3), 193.22 (C-4), 129.91 (C-5), 111.89 (C-6), 165.44 (C-7), 103.91 (C-8), 166.88 (C-9), 115.08 (C-10), 134.51 (C-1′), 128.86 (C-2′, C-6′), 117.9 (C-3′, C-5′), 159.28 (C-4′), 102.28 (C-1″), 74.96 (C-2″), 78.07 (C-3″), 71.45 (C-4″), 78.22 (C-5″), and 62.59 (C-6″).

### 3.6. Quantification of Glycyrrhizic Acid, Glabridin, and Liquiritin by HPLC

The powdered G. glabra root samples (100 mg) were suspended in 10 mL of 50% ethanol and sonicated for 30 min. These suspensions were further incubated at 25 °C for 24 h with shaking at 100 rpm. After centrifugation at 16,000 rpm for 10 min, the samples were used for HPLC analysis. The contents of glycyrrhizic acid, glabridin, and liquiritin were analyzed using a Dacapo DX-C18 column (2 mm × 100 mm, 2.5 µm) under the following conditions: elution with acetonitrile–water linear gradient (10:90 at 0 min to 100:0 at 20 min) containing 0.1% phosphoric acid, 0.3 mL min^−1^ flow rate, 40 °C column temperature, 1.0 µL injection, and detection by a PDA detector (200−360 nm).

Glabridin and glycyrrhizic acid were dissolved in 50% ethanol at 50 µg mL^−1^, 100 µg mL^−1^, 200 µg mL^−1^, and 300 µg mL^−1^, whereas liquiritin was dissolved in methanol at 50 µg mL^−1^, 100 µg mL^−1^, 300 µg mL^−1^, and 500 µg mL^−1^ to obtain the corresponding HPLC standard curves (R^2^ > 0.999).

### 3.7. In-Situ Soil Analysis

The soil pH and compaction were recorded using a conventional pH meter and Yamanaka-type soil hardness tester, respectively. The refractive index ($\sqrt{\varepsilon }$) and VSMC were determined using the SM150T output voltage [26,27] and Equations (1) and (2), respectively:

(1) $$\sqrt{\varepsilon }=1+14.4396\;V-31.2587\;V^{2}+49.0575\;V^{3}-36.5575\;V^{4}+10.7117\;V^{5}$$

(2) $$\mathrm{VSMC}=(\sqrt{\varepsilon }-a_{0})/a_{1}$$

where V is the SM150T output in volts and a_0_ and a_1_ are constant values determined by soil type [28], as shown in Table 4.

### 3.8. Dataset Preparation for Habitat Suitability Zones

**Figure 5:**
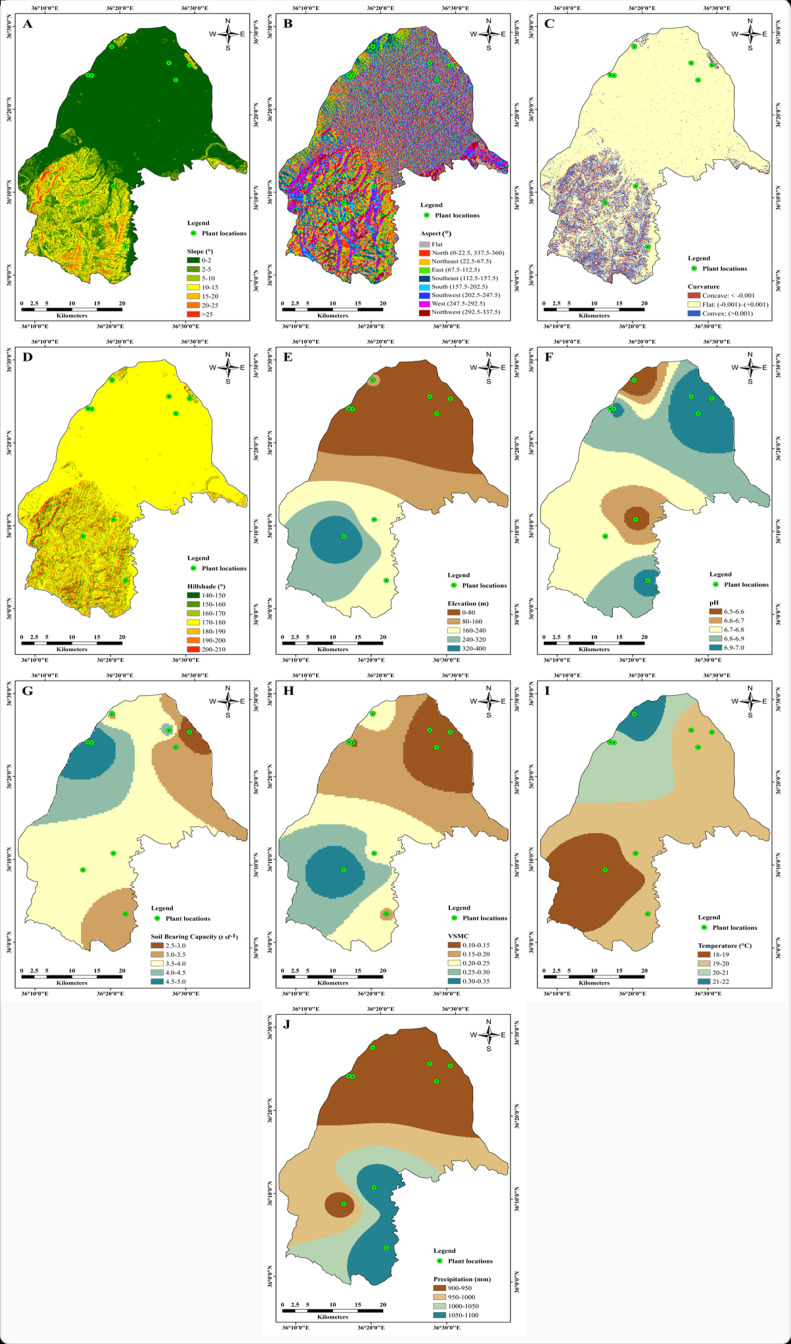
Thematic maps of the study area: (**A**) slope, (**B**) aspect, (**C**) curvature, (**D**) hillshade, (**E**) elevation, (**F**) pH, (**G**) soil bearing capacity, (**H**) VSMC, (**I**) temperature, and (**J**) precipitation.

Different topographical, climatic, and soil datasets for a study area are necessary to identify suitable habitat zones with appropriate GIS-based models [29,30]. In this study, the topographical features (slope, aspect, curvature, hillshade, and elevation), soil conditions (pH, soil bearing capacity, and soil moisture content), and meteorological information (annual mean temperature and annual mean precipitation) were investigated. The topographical features were extracted from the Shuttle Radar Topography Mission–digital elevation model (SRTM–DEM) with 90 m × 90 m resolution (Figure 5A–J). The image was acquired from Jarvis et al. [31]. The slope range (0–52°) was classified into seven classes (Figure 5A). The slope aspect orientation (aspect) map shows the direction and degree of slope for a given surface. The slope aspect of the area was classified into nine classes: flat, north, northeast, east, southeast, south, southwest, west, and northwest (Figure 5B). The south, southwest, and west facing slopes occupied a larger area and had the high possibility of receiving large amounts of sunlight and rainfall. The rate of change of a slope or aspect in a specific direction was represented by the curvature. The curvature regulates the hydrological behavior of the soil and preserves more water in convex slopes after rainfall. The curvature map was classified into three classes: concave (<−0.001), flat (−0.001 to +0.001), and convex (>0.001) (Figure 5C). The hillshade map was also prepared from the SRTM–DEM, and shows topographical forms of highlands by using a color scale divided into seven classes (Figure 5D). The elevation map was divided into five classes ranging from 0 m to 400 m (Figure 5E).

The bioclimatic variables [32], including the annual mean temperature (BIO1), maximum temperature of the warmest month (BIO5), minimum temperature of the coldest month (BIO6), annual precipitation (BIO12), precipitation of the wettest month (BIO13), and precipitation of the driest month (BIO14) for the period 2010–2018, were also considered when preparing the habitat suitability map.

Thematic maps of soil factors and meteorological data were prepared from the 10 plant locations in the study area. The interpolation of these maps was generated by applying the inverse distance weighted (IDW) spatial analyst technique in ArcGIS (Figure 5F–J). Finally, all thematic maps were converted into a raster format with the same resolution of 85 m for further analyses. The HSZM was calculated using the frequency ratio (FR) model and overlay analysis of each thematic map in a GIS environment.

### 3.9. Frequency Ratio (FR) Model

The FR is a commonly used statistical method for the identification and mapping of potential zones. The FR method was adopted in this study to produce habitat suitability zones using multiple thematic spatial datasets. The FR is defined as the ratio of the area of plant locations to the total study area, and is also expressed as the ratio of the probabilities of occurrence to nonoccurrence for a certain plant training subclass (Equation (3)) [29,33].

FR = (points in each subclass/total points)/(class area/total area)(3)

In the GIS environment, all thematic maps were reclassified by their subclasses. For every thematic layer’s subclass, the FR values were calculated, thus obtaining the relative frequency (RF). The prediction ratio (PR) of each thematic map was measured with the geographic coverage of each subclass in the study area. Microsoft Excel and ArcGIS 10.5.1 were used to predict the FR of the thematic factor maps used in this study.

Finally, the HSZM was prepared by adding all of the PR values for each thematic layer, as shown in Equation (4)

HSZM = (1.86 × aspect) + (1.64 × curvature) + (11.11 × elevation) + (9.33 × soil bearing capacity) + (1 × hillshade) + (12.86 × pH) + (11.25 × precipitation) + (13.13 × temperature) + (11.93 × VSMC) + (1.64 × slope) + (14.43 × BIO1) + (4.18 × BIO5) + (6.29 × BIO6) + (7.89 × BIO12) + (5.97 × BIO13) + (7.68 × BIO14)(4)

The HSZM represents the relative environmental potential zones for plant cultivation, conservation, and future investigations. For mapping purposes, the HSZM was reclassified into four main suitability classes (very high, high, moderate, and low) using a natural break classification method.

The accuracy of the final habitat suitability map was assessed using the receiver operator characteristic (ROC) curve and the area under the curve (AUC), which represents the prediction accuracy of the FR model. The ROC curve can be signified as a graphical representation of the tradeoff between the false-positive rate on the X-axis and the true-positive rate on the Y-axis for every possible cutoff value [34,35].

### 3.10. Statistical Tests

All measurements were performed in triplicate. Linear fittings of standard curves were prepared using Microsoft Excel 2013 by plotting the peak area versus concentration. XLSTAT statistical and data analysis solution (Addinsoft, 2020, New York, NY, USA) was used to calculate the Pearson’s correlation, one-way analysis of variance (one-way ANOVA), and multivariate analysis of variance (MANOVA) to estimate the effects of soil, meteorological data, and topographical information on the plant’s chemical contents. XLSTAT was also utilized to assess the prediction accuracy of the FR model using the ROC curve. Any correlation with a *p*-value of < 0.05 was considered to be significant.

## 4. Conclusions

The bioactive compounds of *G. glabra* exhibited a variable response to the effects of environmental and soil parameters. The soil bearing capacity, VSMC, slope, aspect, curvature, and elevation of the study area displayed significant effects on the glycyrrhizic acid and liquiritin contents, which indicates the effect of topographical and soil parameters on the bioactive contents. However, the glabridin content was not affected by the environmental factors or soil conditions, which suggests the influence of other parameters (e.g., genetic variation within the same species). Multiple environmental factors indicated the high potential of *G. glabra* growth and expansion in the Hatay region. Mimicking a similar setting for cultivation purposes of *G. glabra* might prove beneficial, especially if accompanied by a control system for soil aeration and moisture level while maintaining adequate amounts of soil micronutrients. In the future, more investigations will be conducted on the effect of soil properties and environmental factors on the bioactive content of licorice in the Hatay and nearby regions.

## Figures and Tables

**Table 1:** Chemical contents analyzed with HPLC.

Sample Information		Chemical Contents		
ID ^a^	Root Diameter (mm)	Glycyrrhizic Acid (%) ^b^	Glabridin (%) ^b^	Liquiritin (%) ^b^
A-1	8	1.32 ± 0.10	0.16 ± 0.01	0.50 ± 0.03
B-1	17	1.00 ± 0.20	0.15 ± 0.05	0.93 ± 0.10
B-2	16	0.65 ± 0.01	0.10 ± 0.01	0.49 ± 0.01
B-3	10	0.87 ± 0.10	0.09 ± 0.01	0.70 ± 0.10
C-1	11	0.76 ± 0.20	0.04 ± 0.02	0.61 ± 0.20
C-2	13	1.24 ± 0.10	0.07 ± 0.02	1.05 ± 0.10
C-3	13	1.13 ± 0.30	0.09 ± 0.03	0.67 ± 0.20
D-1	11	0.59 ± 0.10	0.03 ± 0.01	0.51 ± 0.10
D-2	10	0.72 ± 0.20	0.05 ± 0.02	0.68 ± 0.10
D-3	6	0.54 ± 0.10	0.02 ± 0.01	0.58 ± 0.10
E-1	17	2.17 ± 0.60	0.24 ± 0.07	0.87 ± 0.20
E-2	17	0.89 ± 0.10	0.07 ± 0.03	0.25 ± 0.03
E-3	20	1.23 ± 0.20	0.05 ± 0.01	0.43 ± 0.10
F-1	16	1.96 ± 0.50	0.10 ± 0.02	1.14 ± 0.20
F-2	11	2.10 ± 0.40	0.07 ± 0.02	1.34 ± 0.30
F-3	20	2.40 ± 0.40	0.11 ± 0.02	1.85 ± 0.20
G-1	15	1.21 ± 0.40	0.09 ± 0.01	1.04 ± 0.40
G-2	12	1.37 ± 0.30	0.11 ± 0.02	0.79 ± 0.10
G-3	27	1.08 ± 0.40	0.04 ± 0.01	1.19 ± 0.50
H-1	7	0.70 ± 0.20	0.09 ± 0.02	0.18 ± 0.04
H-2	10	1.68 ± 0.40	0.19 ± 0.03	0.45 ± 0.10
H-3	12	1.50 ± 0.30	0.08 ± 0.01	0.44 ± 0.10
I-1	8	0.56 ± 0.30	0.18 ± 0.04	0.59 ± 0.30
I-2	16	1.25 ± 0.30	0.11 ± 0.01	1.72 ± 0.30
I-3	11	1.15 ± 0.20	0.19 ± 0.04	1.34 ± 0.20
J-1	15	0.92 ± 0.20	0.10 ± 0.01	0.54 ± 0.10
J-2	14	1.40 ± 0.20	0.06 ± 0.01	1.02 ± 0.10
J-3	15	1.97 ± 0.20	0.31 ± 0.04	1.30 ± 0.10

The experiments were repeated three times, from which the mean and standard deviation (SD) were calculated. ^a^ Uppercase letters in the sample ID indicates the sample location. ^b^ The percentage of content represents the % of dry weight.

**Table 2:** Statistical effects of tested variables on the bioactive contents of *G. glabra*.

Variable	MANOVA Analysis (Wilks’ Test)		ANOVA Analysis					
	Wilks’ Lambda	F Value	Glycyrrhizic Acid Content		Glabridin Content		Liquiritin Content	
			R^2^	F Value	R^2^	F Value	R^2^	F Value
Elevation	0.029 ***	4.152	0.644 **	3.616	0.364	1.146	0.628 *	3.379
Curvature	0.044 ***	4.881	0.569 **	3.778	0.265	1.031	0.608 **	4.424
Hillshade	0.182 ***	4.203	0.064	0.392	0.217	1.598	0.312	2.610
Aspect	0.029 ***	4.152	0.644 **	3.616	0.364	1.146	0.628 *	3.379
Slope	0.060 ***	3.394	0.465	2.065	0.362	1.348	0.568 *	2.468
Soil bearing capacity	0.095 ***	3.861	0.537 **	4.059	0.066	0.246	0.491*	3.381
Soil pH	0.337	1.788	0.207	1.150	0.276	1.675	0.371	2.597
VSMC	0.033 ***	4.634	0.614 **	3.771	0.265	0.857	0.618 **	3.487
Average annual temperature	0.409	1.870	0.292	2.373	0.223	1.647	0.319	2.694
Average annual precipitation	0.585 **	5.666	0.000	0.010	0.162	5.022	0.105	3.047
Climate	0.500 **	3.173	0.337	6.346	0.178	2.710	0.099	1.373

* *p*-value < 0.05, ** *p*-value < 0.01, and *** *p*-value < 0.001.

**Table 3:** Geographical and meteorological data of the collection sites.

Location	Longitude	Latitude	Elevation (m)	Average Temperature (°C) ^a^	Average Precipitation (mm) ^a^	Climate Classification ^b^
A	36°16′0.27″ E	36°24′21.59″ N	75.6	20.4	900	Semi-arid–dry sub-humid
B	36°16′30.41″ E	36°24′19.29″ N	82.5	19.9	900	Semi-arid–dry sub-humid
C	36°19′5.3″ E	36°27′54.3″ N	116.1	21.1	900	Semi-arid–dry sub-humid
D	36°19′5.25″ E	36°27′50.27″ N	125.3	21.1	900	Semi-arid–dry sub-humid
E	36°26′45.21″ E	36°26′3.21″ N	89.4	19.4	900	Semi-humid
F	36°29′30.94″ E	36°25′52.03″ N	88.1	19.4	900	Semi-humid
G	36°27′44.42″ E	36°24′1.09″ N	83.6	19.4	900	Semi-humid
H	36°15′52.8″ E	36°8′56.47″ N	328.0	18.3	900	Humid
I	36°21′42.68″ E	36°3′45.51″ N	198.8	19.4	1100	Humid
J	36°19′50.46″ E	36°11′2.27″ N	164.5	19.4	1100	Humid

^a^ Record of 2019 ^b^ Based on Thornthwaite method for the period 1981–2010 [23] ^a and b^ Acquired data from the Turkish Ministry of Agriculture and Forestry, General Directorate of Meteorology (2020) [22].

**Table 4:** Soil data collected from plant locations.

Location	Soil Bearing Capacity ^a^	pH	SM150T output (V)	$\sqrt{\varepsilon }$ ^b^	VSMC ^c^
A	4.18 ± 0.31	6.83 ± 0.35	0.15 ± 0.01	2.62 ± 0.31	0.17 ± 0.01
B	4.50 ± 1.17	7.00 ± 0.20	0.14 ± 0.05	2.47 ± 0.41	0.15 ± 0.05
C	4.11 ± 1.39	6.53 ± 0.15	0.21 ± 0.03	3.02 ± 0.23	0.22 ± 0.03
D	3.07 ± 0.67	6.70 ± 0.27	0.19 ± 0.01	2.91 ± 0.05	0.21 ± 0.01
E	3.86 ± 0.84	7.03 ± 0.06	0.13 ± 0.03	2.40 ± 0.21	0.14 ± 0.03
F	2.61 ± 0.58	6.93 ± 0.12	0.10 ± 0.01	2.20 ± 0.06	0.12 ± 0.01
G	3.39 ± 1.95	7.00 ± 0.01	0.11 ± 0.02	2.25 ± 0.23	0.12 ± 0.03
H	3.61 ± 0.35	6.83 ± 0.15	0.35 ± 0.12	3.83 ± 0.63	0.33 ± 0.08
I	3.44 ± 0.39	6.93 ± 0.31	0.18 ± 0.07	2.78 ± 0.52	0.19 ± 0.07
J	3.53 ± 0.30	6.80 ± 0.27	0.19 ± 0.04	3.00 ± 0.26	0.22 ± 0.03

All measurements were repeated three times, from which the mean and standard deviation (SD) were calculated. ^a^ Measured in tons per square foot (t sf^−1^). ^b^ The refractive index was calculated from the SM150T output. ^c^ The ratio of the water content in organic soils (m^3^ m^−3^), where a_0_ and a_1_ values are 1.3 and 7.7, respectively.

## Supplementary Materials

The following are available online. The following are attached to this article: Table S1 contains geographic and climate data of collection sites; Table S2 shows Pearson’s correlation; Table S3 contains data used for spatial modeling; Table S4 has soil analysis data; and Table S5 shows in situ photos of plants collected from each location.
