# Supplementary material for: Phytochemical Analysis and Habitat Suitability Mapping of Glycyrrhiza glabra L. Collected in the Hatay Region of Turkey
Source: Molecules. 2020 Nov 25;25(23):5529. doi: 10.3390/molecules25235529 (PMC7728332; doi:10.3390/molecules25235529)
Supplement: Supplementary file 1 [file molecules-25-05529-s001.pdf]

Table S1. Geographic and climate data of collection sites

Variables	Location									
	A	B	C	D	E	F	G	H	I	J
Elevation (m)	75.6	82.5	116.1	125.3	89.4	88.1	83.6	328.0	198.8	164.5
Curvature	0.11	−0.11	−0.11	0.22	0.67	0	−0.22	−0.78	2.22	−0.11
Hillshade (°)	0	0	0	0	92	0	0	112	110	86
Aspect (°)	135.0	213.7	127.9	187.6	255.9	225	153.4	6.5	7.2	253.7
Slope (°)	0.7	0.9	2.9	8.5	4.8	1.9	1.9	21	15.7	13.4
Average temp. (°C)*	20.4	19.9	21.1	21.1	19.4	19.4	19.4	18.3	19.4	19.4
Maximum temp. (°C)*	37.1	37.1	37.1	37.1	37.1	37.1	37.1	39.6	39.6	37.1
Minimum temp. (°C)*	2.5	2.5	2.5	2.5	2.5	2.5	2.5	−2.0	−2.0	−2.0
Maximum daily rainfall (mm)*	62.5	62.5	12.5	12.5	12.5	12.5	12.5	62.5	62.5	62.5
Average areal precipitation (mm)*	900	900	900	900	900	900	900	900	1100	1100
Precipitation efficiency index**	−9.5	−9.5	−9.5	−9.5	10.5	10.5	10.5	30.5	30.5	30.5
Average annual temp. (°C)***	19.4	19.4	19.5	19.5	19.5	19.4	19.5	18.0	19.1	19.2
Average max. temp. of warmest month (°C)***	32.2	32.2	33.0	33.0	32.6	32.9	32.8	32.3	33.8	32.9
Average min. temp. of coldest month (°C)***	6.1	6.1	6.0	6.0	5.5	5.3	5.4	4.1	5.0	5.5
Average annual precipitation (mm)***	855	855	813	813	731	702	720	890	778	836
Average precipitation of wettest month (mm)***	139	139	132	132	123	120	122	172	145	151
Average precipitation of driest month (mm)***	9	9	8	8	5	4	5	5	4	7

* Recorded in 2019 (Turkish Ministry of Agriculture and Forestry, General Directorate of Meteorology. https://www.mgm.gov.tr/. (Accessed on Jan.11th 2020))
** Climate classification based on Thronthwaite method for the period 1981–2010 (Thornthwaite 1948)
***Bioclimatic variables for the period 2010-2018 (WorldClim, Bioclimatic variables. https://www.worldclim.org/data/bioclim.html. (Accessed on May 1st 2020))

Table S2. Pearson`s correlation

Variables	Elevation (m)	Curvature	Hillshade (°)	Aspect (°)	Slope (°)	glycyrrhizic acid (%)	glabridin (%)	liquiritin (%)	Soil bearing capacity (t sf^{-1})	pH	VSMC (m^3 m^{-3})	root diameter (cm)	Average temp. (°C)*	Maximum temp. (°C)*	Minimum temp. (°C)*	Maximum daily rainfall (mm)*	Average areal precipitation (mm)*	Precipitation efficiency index**	Average annual temp. (°C)***	Average max. temp. of warmest month (°C)***	Average min. temp. of coldest month (°C)***	Average annual precipitation (mm)***	Average precipitation of wettest month (mm)***	Average precipitation of driest month (mm)***
Elevation (m)	**1**	-0.065	**0.714**	**-0.726**	**0.940**	-0.057	0.196	-0.239	-0.061	-0.163	**0.922**	**-0.391**	**-0.552**	**0.857**	**-0.829**	**0.554**	0.292	**0.670**	**-0.954**	0.038	**-0.829**	**0.574**	**0.901**	-0.296
Curvature	-0.065	**1**	0.357	-0.242	0.148	-0.144	0.211	0.302	-0.112	0.235	-0.287	-0.010	0.071	0.349	-0.218	0.113	**0.571**	0.225	0.237	**0.765**	0.022	-0.325	-0.163	-0.342
Hillshade (°)	**0.714**	0.357	**1**	**-0.394**	**0.822**	0.072	**0.422**	-0.145	0.042	0.217	**0.515**	-0.056	**-0.663**	**0.711**	**-0.823**	**0.525**	**0.576**	**0.839**	**-0.644**	0.191	**-0.709**	0.220	**0.629**	**-0.493**
Aspect (°)	**-0.726**	-0.242	**-0.394**	**1**	**-0.613**	0.218	-0.106	0.072	0.076	0.069	**-0.632**	**0.412**	0.273	**-0.887**	**0.533**	**-0.384**	-0.162	-0.371	**0.665**	-0.288	**0.621**	-0.339	**-0.582**	0.304
Slope (°)	**0.940**	0.148	**0.822**	**-0.613**	**1**	-0.092	0.251	-0.170	-0.093	-0.142	**0.816**	-0.358	**-0.513**	**0.820**	**-0.914**	**0.571**	**0.529**	**0.765**	**-0.829**	0.232	**-0.758**	**0.488**	**0.846**	-0.329
glycyrrhizic acid (%)	-0.057	-0.144	0.072	0.218	-0.092	**1**	**0.454**	**0.587**	**-0.461**	0.290	-0.195	0.338	**-0.397**	-0.089	-0.013	-0.139	-0.020	0.301	-0.067	-0.069	-0.275	**-0.397**	-0.162	**-0.467**
glabridin (%)	0.196	0.211	**0.422**	-0.106	0.251	**0.454**	**1**	0.215	0.145	0.233	0.077	-0.025	-0.364	0.263	**-0.406**	**0.455**	**0.402**	**0.410**	-0.206	0.029	-0.233	0.114	0.281	-0.165
liquiritin (%)	-0.239	0.302	-0.145	0.072	-0.170	**0.587**	0.215	**1**	**-0.496**	0.176	**-0.424**	**0.408**	-0.042	-0.052	-0.024	-0.098	0.324	0.177	0.265	**0.486**	0.018	**-0.490**	-0.322	-0.358
Soil bearing capacity (t sf^{-1})	-0.061	-0.112	0.042	0.076	-0.093	**-0.461**	0.145	**-0.496**	**1**	-0.040	0.133	-0.063	0.144	-0.098	0.033	**0.473**	-0.043	-0.302	0.038	**-0.508**	0.355	**0.616**	0.287	**0.689**
pH	-0.163	0.235	0.217	0.069	-0.142	0.290	0.233	0.176	-0.040	**1**	**-0.443**	**0.449**	**-0.648**	0.126	0.045	0.070	-0.034	0.322	-0.004	-0.148	-0.308	**-0.493**	-0.230	**-0.578**
VSMC (m^3 m^{-3})	**0.922**	-0.287	**0.515**	**-0.632**	**0.816**	-0.195	0.077	**-0.424**	0.133	**-0.443**	**1**	**-0.489**	-0.286	**0.661**	**-0.660**	**0.483**	0.106	**0.385**	**-0.873**	-0.145	**-0.599**	**0.772**	**0.905**	0.048
root diameter (cm)	**-0.391**	-0.010	-0.056	**0.412**	-0.358	0.338	-0.025	**0.408**	-0.063	**0.449**	**-0.489**	**1**	-0.193	-0.332	0.232	-0.265	-0.039	0.063	0.313	-0.055	0.081	**-0.502**	**-0.424**	-0.249
Average temp. (°C)*	**-0.552**	0.071	**-0.663**	0.273	**-0.513**	**-0.397**	-0.364	-0.042	0.144	**-0.648**	-0.286	-0.193	**1**	**-0.558**	**0.582**	**-0.439**	-0.208	**-0.816**	**0.690**	0.197	**0.851**	0.010	**-0.459**	**0.681**
Maximum temp. (°C)*	**0.857**	0.349	**0.711**	**-0.887**	**0.820**	-0.089	0.263	-0.052	-0.098	0.126	**0.661**	-0.332	**-0.558**	**1**	**-0.759**	**0.561**	0.364	**0.657**	**-0.803**	0.279	**-0.806**	0.331	**0.710**	**-0.487**
Minimum temp. (°C)*	**-0.829**	-0.218	**-0.823**	**0.533**	**-0.914**	-0.013	**-0.406**	-0.024	0.033	0.045	**-0.660**	0.232	**0.582**	**-0.759**	**1**	**-0.739**	**-0.759**	**-0.866**	**0.726**	-0.291	**0.692**	**-0.443**	**-0.825**	0.330
Maximum daily rainfall (mm)*	**0.554**	0.113	**0.525**	**-0.384**	**0.571**	-0.139	**0.455**	-0.098	**0.473**	0.070	**0.483**	-0.265	**-0.439**	**0.561**	**-0.739**	**1**	**0.561**	**0.475**	**-0.548**	-0.118	-0.334	**0.695**	**0.802**	0.125
Average areal precipitation (mm)*	0.292	**0.571**	**0.576**	-0.162	**0.529**	-0.020	**0.402**	0.324	-0.043	-0.034	0.106	-0.039	-0.208	0.364	**-0.759**	**0.561**	**1**	**0.657**	-0.107	**0.629**	-0.184	0.100	0.357	-0.203
Precipitation efficiency index**	**0.670**	0.225	**0.839**	-0.371	**0.765**	0.301	**0.410**	0.177	-0.302	0.322	**0.385**	0.063	**-0.816**	**0.657**	**-0.866**	**0.475**	**0.657**	**1**	**-0.638**	0.292	**-0.823**	0.007	**0.541**	**-0.703**
Average annual temp. (°C)***	**-0.954**	0.237	**-0.644**	**0.665**	**-0.829**	-0.067	-0.206	0.265	0.038	-0.004	**-0.873**	0.313	**0.690**	**-0.803**	**0.726**	**-0.548**	-0.107	**-0.638**	**1**	0.200	**0.880**	**-0.544**	**-0.871**	0.328
Average max. temp. of warmest month (°C)***	0.038	**0.765**	0.191	-0.288	0.232	-0.069	0.029	**0.486**	**-0.508**	-0.148	-0.145	-0.055	0.197	0.279	-0.291	-0.118	**0.629**	0.292	0.200	**1**	-0.039	**-0.383**	-0.164	**-0.393**
Average min. temp. of coldest month (°C)***	**-0.829**	0.022	**-0.709**	**0.621**	**-0.758**	-0.275	-0.233	0.018	0.355	-0.308	**-0.599**	0.081	**0.851**	**-0.806**	**0.692**	-0.334	-0.184	**-0.823**	**0.880**	-0.039	**1**	-0.096	**-0.603**	**0.732**
Average annual precipitation (mm)***	**0.574**	-0.325	0.220	-0.339	**0.488**	**-0.397**	0.114	**-0.490**	**0.616**	**-0.493**	**0.772**	**-0.502**	0.010	0.331	**-0.443**	**0.695**	0.100	0.007	**-0.544**	**-0.383**	-0.096	**1**	**0.830**	**0.588**
Average precipitation of wettest month (mm)***	**0.901**	-0.163	**0.629**	**-0.582**	**0.846**	-0.162	0.281	-0.322	0.287	-0.230	**0.905**	**-0.424**	**-0.459**	**0.710**	**-0.825**	**0.802**	0.357	**0.541**	**-0.871**	-0.164	**-0.603**	**0.830**	**1**	0.050
Average precipitation of driest month (mm)***	-0.296	-0.342	**-0.493**	0.304	-0.329	**-0.467**	-0.165	-0.358	**0.689**	**-0.578**	0.048	-0.249	**0.681**	**-0.487**	0.330	0.125	-0.203	**-0.703**	0.328	**-0.393**	**0.732**	**0.588**	0.050	**1**

Values in bold are different from 0 with a significance level alpha=0.05

* Recorded in 2019 (Turkish Ministry of Agriculture and Forestry, General Directorate of Meteorology. https://www.mgm.gov.tr/. (Accessed on Jan.11th 2020))

** Climate classification based on Thronthwaite method for the period 1981–2010 (Thornthwaite 1948)

***Bioclimatic variables for the period 2010-2018 (WorldClim, Bioclimatic variables. https://www.worldclim.org/data/bioclim.html. (Accessed on May 1st 2020))

p-values (Pearson):

Variables	Elevation (m)	Curvature	Hillshade (°)	Aspect (°)	Slope (°)	glycyrrhizic acid (%)	glabridin (%)	liquiritin (%)	Soil bearing capacity (t sf^{-1})	pH	VSMC (m^3 m^{-3})	root diameter (cm)	Average temp. (°C)*	Maximum temp. (°C)*	Minimum temp. (°C)*	Maximum daily rainfall (mm)*	Average areal precipitation (mm)*	Precipitation efficiency index**	Average annual temp. (°C)***	Average max. temp. of warmest month (°C)***	Average min. temp. of coldest month (°C)***	Average annual precipitation (mm)***	Average precipitation of wettest month (mm)***	Average precipitation of driest month (mm)***
Elevation (m)	**0**	0.743	**<0.0001**	**<0.0001**	**<0.0001**	0.771	0.317	0.220	0.759	0.409	**<0.0001**	**0.039**	**0.002**	**<0.0001**	**<0.0001**	**0.002**	0.132	**<0.0001**	**<0.0001**	0.849	**<0.0001**	**0.001**	**<0.0001**	0.127
Curvature	0.743	**0**	0.062	0.215	0.453	0.464	0.280	0.118	0.572	0.229	0.139	0.958	0.720	0.069	0.266	0.567	**0.001**	0.250	0.224	**<0.0001**	0.913	0.091	0.407	0.075
Hillshade (°)	**<0.0001**	0.062	**0**	**0.038**	**<0.0001**	0.715	**0.025**	0.461	0.833	0.268	**0.005**	0.777	**0.000**	**<0.0001**	**<0.0001**	**0.004**	**0.001**	**<0.0001**	**0.000**	0.330	**<0.0001**	0.260	**0.000**	**0.008**
Aspect (°)	**<0.0001**	0.215	**0.038**	**0**	**0.001**	0.266	0.590	0.716	0.702	0.727	**0.000**	**0.029**	0.160	**<0.0001**	**0.003**	**0.044**	0.409	0.052	**0.000**	0.137	**0.000**	0.078	**0.001**	0.116
Slope (°)	**<0.0001**	0.453	**<0.0001**	**0.001**	**0**	0.643	0.197	0.388	0.638	0.469	**<0.0001**	0.062	**0.005**	**<0.0001**	**<0.0001**	**0.001**	**0.004**	**<0.0001**	**<0.0001**	0.236	**<0.0001**	**0.008**	**<0.0001**	0.087
glycyrrhizic acid (%)	0.771	0.464	0.715	0.266	0.643	**0**	**0.015**	**0.001**	**0.014**	0.135	0.321	0.079	**0.036**	0.654	0.948	0.481	0.920	0.119	0.736	0.729	0.156	**0.036**	0.410	**0.012**
glabridin (%)	0.317	0.280	**0.025**	0.590	0.197	**0.015**	**0**	0.272	0.461	0.232	0.696	0.901	0.057	0.177	**0.032**	**0.015**	**0.034**	**0.030**	0.294	0.884	0.232	0.565	0.148	0.400
liquiritin (%)	0.220	0.118	0.461	0.716	0.388	**0.001**	0.272	**0**	**0.007**	0.370	**0.025**	**0.031**	0.832	0.791	0.904	0.621	0.093	0.366	0.173	**0.009**	0.928	**0.008**	0.094	0.061
Soil bearing capacity (t sf^{-1})	0.759	0.572	0.833	0.702	0.638	**0.014**	0.461	**0.007**	**0**	0.840	0.500	0.749	0.466	0.619	0.869	**0.011**	0.827	0.118	0.847	**0.006**	0.064	**0.000**	0.139	**<0.0001**
pH	0.409	0.229	0.268	0.727	0.469	0.135	0.232	0.370	0.840	**0**	**0.018**	**0.016**	**0.000**	0.524	0.820	0.722	0.863	0.095	0.983	0.452	0.111	**0.008**	0.239	**0.001**
VSMC (m^3 m^{-3})	**<0.0001**	0.139	**0.005**	**0.000**	**<0.0001**	0.321	0.696	**0.025**	0.500	**0.018**	**0**	**0.008**	0.140	**0.000**	**0.000**	**0.009**	0.592	**0.043**	**<0.0001**	0.463	**0.001**	**<0.0001**	**<0.0001**	0.807
root diameter (cm)	**0.039**	0.958	0.777	**0.029**	0.062	0.079	0.901	**0.031**	0.749	**0.016**	**0.008**	**0**	0.325	0.084	0.235	0.172	0.843	0.749	0.104	0.782	0.684	**0.006**	**0.025**	0.202
Average temp. (°C)*	**0.002**	0.720	**0.000**	0.160	**0.005**	**0.036**	0.057	0.832	0.466	**0.000**	0.140	0.325	**0**	**0.002**	**0.001**	**0.019**	0.288	**<0.0001**	**<0.0001**	0.315	**<0.0001**	0.961	**0.014**	**<0.0001**
Maximum temp. (°C)*	**<0.0001**	0.069	**<0.0001**	**<0.0001**	**<0.0001**	0.654	0.177	0.791	0.619	0.524	**0.000**	0.084	**0.002**	**0**	**<0.0001**	**0.002**	0.057	**0.000**	**<0.0001**	0.150	**<0.0001**	0.086	**<0.0001**	**0.009**
Minimum temp. (°C)*	**<0.0001**	0.266	**<0.0001**	**0.003**	**<0.0001**	0.948	**0.032**	0.904	0.869	0.820	**0.000**	0.235	**0.001**	**<0.0001**	**0**	**<0.0001**	**<0.0001**	**<0.0001**	**<0.0001**	0.133	**<0.0001**	**0.018**	**<0.0001**	0.087
Maximum daily rainfall (mm)*	**0.002**	0.567	**0.004**	**0.044**	**0.001**	0.481	**0.015**	0.621	**0.011**	0.722	**0.009**	0.172	**0.019**	**0.002**	**<0.0001**	**0**	**0.002**	**0.011**	**0.003**	0.549	0.082	**<0.0001**	**<0.0001**	0.526
Average areal precipitation (mm)*	0.132	**0.001**	**0.001**	0.409	**0.004**	0.920	**0.034**	0.093	0.827	0.863	0.592	0.843	0.288	0.057	**<0.0001**	**0.002**	**0**	**0.000**	0.589	**0.000**	0.348	0.613	0.062	0.301
Precipitation efficiency index**	**<0.0001**	0.250	**<0.0001**	0.052	**<0.0001**	0.119	**0.030**	0.366	0.118	0.095	**0.043**	0.749	**<0.0001**	**0.000**	**<0.0001**	**0.011**	**0.000**	**0**	**0.000**	0.132	**<0.0001**	0.973	**0.003**	**<0.0001**
Average annual temp. (°C)***	**<0.0001**	0.224	**0.000**	**0.000**	**<0.0001**	0.736	0.294	0.173	0.847	0.983	**<0.0001**	0.104	**<0.0001**	**<0.0001**	**<0.0001**	**0.003**	0.589	**0.000**	**0**	0.309	**<0.0001**	**0.003**	**<0.0001**	0.089
Average max. temp. of warmest month (°C)***	0.849	**<0.0001**	0.330	0.137	0.236	0.729	0.884	**0.009**	**0.006**	0.452	0.463	0.782	0.315	0.150	0.133	0.549	**0.000**	0.132	0.309	**0**	0.844	**0.044**	0.404	**0.038**
Average min. temp. of coldest month (°C)***	**<0.0001**	0.913	**<0.0001**	**0.000**	**<0.0001**	0.156	0.232	0.928	0.064	0.111	**0.001**	0.684	**<0.0001**	**<0.0001**	**<0.0001**	0.082	0.348	**<0.0001**	**<0.0001**	0.844	**0**	0.626	**0.001**	**<0.0001**
Average annual precipitation (mm)***	**0.001**	0.091	0.260	0.078	**0.008**	**0.036**	0.565	**0.008**	**0.000**	**0.008**	**<0.0001**	**0.006**	0.961	0.086	**0.018**	**<0.0001**	0.613	0.973	**0.003**	**0.044**	0.626	**0**	**<0.0001**	**0.001**
Average precipitation of wettest month (mm)***	**<0.0001**	0.407	**0.000**	**0.001**	**<0.0001**	0.410	0.148	0.094	0.139	0.239	**<0.0001**	**0.025**	**0.014**	**<0.0001**	**<0.0001**	**<0.0001**	0.062	**0.003**	**<0.0001**	0.404	**0.001**	**<0.0001**	**0**	0.802
Average precipitation of driest month (mm)***	0.127	0.075	**0.008**	0.116	0.087	**0.012**	0.400	0.061	**<0.0001**	**0.001**	0.807	0.202	**<0.0001**	**0.009**	0.087	0.526	0.301	**<0.0001**	0.089	**0.038**	**<0.0001**	**0.001**	0.802	**0**

Table S3. Data used for spatial modeling

Sl. No.	Variables	Reclassify		Classes and subclass				Plant Pixels			FR	RF	RF (non%)	RF (INT)	Min(RF)	Max(RF)	Max(RF) - Min(RF)	[Max-Min]*Min(RF)*	PR	PR Value
		Class	no. classes	OID	Value	Class Pixel	%Subclass Pixel	Area	Pixels	%Pixels										PR Value
1	Aspect (°)	(−)1–40	1	0	1	259477	12.83	996300	1107	14.08	1.10	0.12	12.28	12						
		40–80	2	1	2	231529	11.45	415800	462	5.87	0.51	0.06	5.74	5						
		80–120	3	2	3	234080	11.57	574200	638	8.11	0.70	0.08	7.84	7						
		120–160	4	3	4	258309	12.77	1125900	1251	15.91	1.25	0.14	13.93	13						
		160–200	5	4	5	247161	12.22	1442700	1603	20.38	1.67	0.19	18.66	18						
		200–240	6	5	6	190569	9.42	721800	802	10.20	1.08	0.12	12.11	12						
		240–280	7	6	7	171593	8.48	700200	778	9.89	1.17	0.13	13.05	13						
		280–320	8	7	8	222734	11.01	562500	625	7.95	0.72	0.08	8.07	8						
		320–360	9	8	9	207199	10.24	539100	599	7.62	0.74	0.08	8.32	8						
	Total					**2022651**			**7865**		**8.94**	**1.00**	**100.00**		**0.06**	**0.19**	**0.13**	**0.07**	**1.87**	**1.86**
2	Curvature	(−)25–(−)1.5	1	0	1	139370	6.90	351000	390	4.96	0.72	0.27	26.62	26						
		(−)1.5–1.5	2	1	2	1741293	86.15	6256800	6952	88.39	1.03	0.38	37.97	37						
		1.5–25	3	2	3	140482	6.95	470700	523	6.65	0.96	0.35	35.41	35						
	**Total**					**2021145**			**7865**		**2.70**	**1.00**	**100.00**		**0.27**	**0.38**	**0.11**	**0.07**	**1.64**	**1.64**
3	Elevation (m)	75–130	1	0	1	1178213	58.25	4721400	5246	66.70	1.15	0.11	10.84	10						
		130–185	2	1	2	322827	15.96	788400	876	11.14	0.70	0.07	6.61	6						
		185–240	3	2	3	381501	18.86	785700	873	11.10	0.59	0.06	5.57	5						
		240–295	4	3	4	112594	5.57	0	0	0.00	0.00	0.00	0.00	0						
		295–350	5	4	5	27516	1.36	783000	870	11.06	8.13	0.77	76.98	76						
	**Total**					**2022651**			**7865**		**10.56**	**1.00**	**100.00**		**0.00**	**0.77**	**0.77**	**0.07**	**11.11**	**11.11**
4	Soil bearing capacity (t sf⁻¹)	2.5–3.0	1	0	1	21852	1.08	785700	873	11.10	10.27	0.68	68.47	68						
		3.0–3.5	2	1	2	392009	19.38	1900800	2112	26.85	1.39	0.09	9.23	9						
		3.5–4.0	3	2	3	1458332	72.10	2933100	3259	41.44	0.57	0.04	3.83	3						
		4.0–4.5	4	3	4	150458	7.44	1458900	1621	20.61	2.77	0.18	18.47	18						
	**Total**					**2022651**			**7865**		**15.01**	**1.00**	**100.00**		**0.04**	**0.68**	**0.65**	**0.07**	**9.33**	**9.33**
5	Hillshade (°)	0–23	1	0	1	1142838	56.50	4176000	4640	59.00	1.04	0.21	20.68	20						
		23–68	2	1	2	131839	6.52	504000	560	7.12	1.09	0.22	21.64	21						
		68–111	3	2	3	154800	7.65	605700	673	8.56	1.12	0.22	22.14	22						
		111–152	4	3	4	219283	10.84	787500	875	11.13	1.03	0.20	20.32	20						
		152–181	5	4	5	373891	18.49	1005300	1117	14.20	0.77	0.15	15.22	15						
	**Total**					**2022651**			**7865**		**5.05**	**1.00**	**100.00**		**0.15**	**0.22**	**0.07**	**0.07**	**1.00**	**1**
6	pH	6.5–6.6	1	0	1	390	0.02	336600	374	4.76	246.58	0.89	89.19	89						
		6.6–6.7	2	1	2	54997	2.72	569700	633	8.05	2.96	0.01	1.07	1						
		6.7–6.8	3	2	3	248041	12.27	788400	876	11.14	0.91	0.00	0.33	0						
		6.8–6.9	4	3	4	1263009	62.45	1460700	1623	20.64	0.33	0.00	0.12	0						
		6.9–7.0	5	4	5	446397	22.07	3138300	3487	44.34	2.01	0.01	0.73	0						
		7.0–7.1	6	5	6	9475	0.47	784800	872	11.09	23.66	0.09	8.56	8						
	**Total**					**2022309**			**7865**		**276.45**	**1.00**	**100.00**		**0.00**	**0.89**	**0.89**	**0.07**	**12.86**	**12.86**
7	Average areal precipitation (mm)*	900–940	1	0	1	1221090	60.37	5504400	6116	77.76	1.29	0.22	22.04	22						
		940–980	2	1	2	453773	22.43	0	0	0.00	0.00	0.00	0.00	0						
		980–1020	3	2	3	133972	6.62	0	0	0.00	0.00	0.00	0.00	0						
		1020–1060	4	3	4	115083	5.69	0	0	0.00	0.00	0.00	0.00	0						
		1060–1100	5	4	5	98733	4.88	1574100	1749	22.24	4.56	0.78	77.96	77						
	**Total**					**2022651**			**7865**		**5.84**	**1.00**	**100.00**		**0.00**	**0.78**	**0.78**	**0.07**	**11.25**	**11.25**
8	Average temp. (°C)*	18–19	1	0	1	164026	8.11	783000	870	11.06	1.36	0.04	3.54	3						
		19–20	2	1	2	1440412	71.21	4518000	5020	63.83	0.90	0.02	2.33	2						
		20–21	3	2	3	410943	20.32	871200	968	12.31	0.61	0.02	1.57	1						
		21–22	4	3	4	7270	0.36	906300	1007	12.80	35.62	0.93	92.55	92						
	**Total**					**2022651**			**7865**		**38.49**	**1.00**	**100.00**		**0.02**	**0.93**	**0.91**	**0.07**	**13.13**	**13.13**
9	VSMC ($m^3 m^{-3}$)	0.10–0.18	1	0	1	1071748	52.99	3815100	4239	53.90	1.02	0.09	8.84	8						
		0.18–0.25	2	1	2	727666	35.98	2480400	2756	35.04	0.97	0.08	8.47	8						
		0.25–0.33	3	2	3	199709	9.87	0	0	0.00	0.00	0.00	0.00	0						
		0.33–0.40	4	3	4	23528	1.16	783000	870	11.06	9.51	0.83	82.69	82						
	**Total**					**2022651**			**7865**		**11.50**	**1.00**	**100.00**		**0.00**	**0.83**	**0.83**	**0.07**	**11.94**	**11.93**

10	Slope (°)	0–3	1	0	1	1268826	62.73		4706100	5229	66.48	1.06	0.18	18.32	18						
		3–7	2	1	2	231927	11.47		691200	768	9.76	0.85	0.15	14.72	14						
		7–12	3	2	3	196895	9.73		670500	745	9.47	0.97	0.17	16.82	16						
		12–17	4	3	4	150517	7.44		568800	632	8.04	1.08	0.19	18.66	18						
		17–24	5	4	5	109658	5.42		324000	360	4.58	0.84	0.15	14.59	14						
		24–32	6	5	6	47809	2.36		92700	103	1.31	0.55	0.10	9.58	9						
		32–64	7	6	7	17019	0.84		25200	28	0.36	0.42	0.07	7.31	7						
	**Total**					**2022651**				**7865**		**5.79**	**1.00**	**100.00**		**0.07**	**0.19**	**0.11**	**0.07**	**1.64**	**1.64**
11	Average annual mean temperature (°C)**	12–14	1	0	1	1647	0.08		0	0	0.00	0.00	0.00	0.00	0						
		14–16	2	1	2	11990	0.59		0	0	0.00	0.00	0.00	0.00	0						
		16–18	3	2	3	269896	13.34		0	0	0.00	0.00	0.00	0.00	0						
		18–20	4	3	4	1739118	85.98		7078500	7865	100.00	1.16	1.00	100.00	100						
	**Total**					**2022651**				**7865**		**1.16**	**1.00**	**100.00**		**0.00**	**1.00**	**1.00**	**0.07**	**14.44**	**14.43**
12	Average max. temperature of warmest month (°C)**	29.5–31.8	1	0	1	106336	5.26		0	0	0.00	0.00	0.00	0.00	0						
		31.8–32.4	2	1	2	575022	28.43		1836000	2040	25.94	0.91	0.22	22.15	22						
		32.4–32.99	3	2	3	591537	29.25		3550500	3945	50.16	1.72	0.42	41.63	41						
		32.99–33.9	4	3	4	544024	26.90		993600	1104	14.04	0.52	0.13	12.67	12						
		33.9–34.8	5	4	5	205732	10.17		698400	776	9.87	0.97	0.24	23.55	23						
	**Total**					**2022651**				**7865**		**4.12**	**1.00**	**100.00**		**0.13**	**0.42**	**0.29**	**0.07**	**4.18**	**4.18**
13	Average min. temperature of coldest month (°C)**	(−)1.6–0.2	1	0	1	3941	0.19		0	0	0.00	0.00	0.00	0.00	0						
		0.2–1.3	2	1	2	2708	0.13		0	0	0.00	0.00	0.00	0.00	0						
		1.3–2.2	3	2	3	11450	0.57		0	0	0.00	0.00	0.00	0.00	0						
		2.2–2.9	4	3	4	36359	1.80		0	0	0.00	0.00	0.00	0.00	0						
		2.9–3.6	5	4	5	103930	5.14		0	0	0.00	0.00	0.00	0.00	0						
		3.6–4.3	6	5	6	201169	9.95		377100	419	5.33	0.54	0.16	15.89	15						
		4.3–4.9	7	6	7	270508	13.37		483300	537	6.83	0.51	0.15	15.15	15						
		4.9–5.4	8	7	8	439229	21.72		1314000	1460	18.56	0.85	0.25	25.36	25						
		5.4–6.2	9	8	9	953357	47.13		4904100	5449	69.28	1.47	0.44	43.61	43						
	**Total**					**2022651**	.			**7865**		**3.37**	**1.00**	**100.00**		**0.00**	**0.44**	**0.44**	**0.07**	**6.29**	**6.29**
14	Average annual precipitation (mm)**	550–650	1	0	1	179515	8.88		0	0	0.00	0.00	0.00	0.00	0						
		650–750	2	1	2	497152	24.58		0	0	0.00	0.00	0.00	0.00	0						
		750–850	3	2	3	571596	28.26		2356200	2618	33.29	1.18	0.21	21.03	21						
		850–950	4	3	4	600861	29.71		2862900	3181	40.45	1.36	0.24	24.31	24						
		950–1050	5	4	5	173527	8.58		1859400	2066	26.27	3.06	0.55	54.66	54						
	**Total**					**2022651**				**7865**		**5.60**	**1.00**	**100.00**		**0.00**	**0.55**	**0.55**	**0.07**	**7.89**	**7.89**
15	Average precipitation of wettest month (mm)**	100–125	1	0	1	597932	29.56		2356200	2618	33.29	1.13	0.34	34.20	34						
		125–150	2	1	2	661037	32.68		3150900	3501	44.51	1.36	0.41	41.37	41						
		150–175	3	2	3	558164	27.60		1571400	1746	22.20	0.80	0.24	24.43	24						
		175–200	4	3	4	193456	9.56		0	0	0.00	0.00	0.00	0.00	0						
		200–225	5	4	5	12062	0.60		0	0	0.00	0.00	0.00	0.00	0						
	**Total**					**2022651**				**7865**		**3.29**	**1.00**	**100.00**		**0.00**	**0.41**	**0.41**	**0.07**	**5.97**	**5.97**
16	Average precipitation of driest month (mm)**	1–3	1	0	1	134516	6.65		0	0	0.00	0.00	0.00	0.00	0						
		3–6	2	1	2	1144927	56.61		4407300	4897	62.26	1.10	0.47	46.79	46						
		6–9	3	2	3	610285	30.17		2671200	2968	37.74	1.25	0.53	53.21	53						
		9–12	4	3	4	132923	6.57		0	0	0.00	0.00	0.00	0.00	0						
	**Total**					**2022651**				**7865**		**2.35**	**1.00**	**100.00**		**0.00**	**0.53**	**0.53**	**0.07**	**7.68**	**7.68**

* Recorded in 2019 (Turkish Ministry of Agriculture and Forestry, General Directorate of Meteorology. https://www.mgm.gov.tr/. (Accessed on Jan.11th 2020))

**Bioclimatic variables for the period 2010-2018 (WorldClim, Bioclimatic variables. https://www.worldclim.org/data/bioclim.html. (Accessed on May 1st 2020))

Table S4. Soil Analysis

Location	pH	EC [a]	N [b]	$(NO^{3-})^b$	(P)[b]	(K)[b]	$[Ca(OH)_2]^b$	(Mg)[b]	(Mn)[c]	(Fe)[c]	(Cu)[c]	(Zn)[c]	(B)[c]
Point - 1*	8.4	0.29	5.3	4.6	7.4	70	797	201	5.9	2.3	0.23	0.29	0.73
Point - 2*	8.5	0.31	4.6	4.3	5.0	55	785	203	5.7	2.3	0.21	0.21	0.84
Point - 3*	8.5	0.29	5.1	5.3	8.8	127	686	159	4.9	3.5	0.26	0.30	1.60
Point - 4**	8.6	0.28	7.0	3.2	16.0	98	782	195	4.3	1.2	0.38	0.37	1.90
Point - 5**	8.7	0.26	6.8	1.6	10.0	63	775	218	4.5	1.3	0.45	0.36	1.80
Standard value (Japan standard) ***	6.0-6.5	0.10-0.30	0.3-1.5	0.7-3.5	20-60	15-40	200-400	35-70	7.0-20.0	15-100	1.0-3.5	10.0-40.0	0.7-2.5

* at 36° 24' 8.36" (Longitude) and 36° 21' 25.76" (Latitude)

**at 36° 26' 22.75" (Longitude) and 36° 22' 33.42" (Latitude)

*** Source: (https://n-seikaken.co.jp/soil/check-shindan.html)

[a] Unit is mS/cm

[b] Unit is mg/100g

[c] Unit is mg/Kg

Table S5. Photographs of *G. glabra* in situ (at the collection sites).

Location	Longitude	Latitude	Elevation (m)
A	36° 16' 0.27" E	36° 24' 21.59" N	75.6
B	36° 16' 30.41" E	36° 24' 19.29" N	82.5
C	36° 19' 5.3" E	36° 27' 54.3" N	116.1
D	36° 19' 5.25" E	36° 27' 50.27" N	125.3
E	36° 26' 45.21" E	36° 26' 3.21" N	89.4
F	36° 29' 30.94" E	36° 25' 52.03" N	88.1
G	36° 27' 44.42" E	36° 24' 1.09" N	83.6
H	36° 15' 52.8" E	36° 8' 56.47" N	328.0
I	36° 21' 42.68" E	36° 3' 45.51" N	198.8
J	36° 19' 50.46" E	36° 11' 2.27" N	164.5

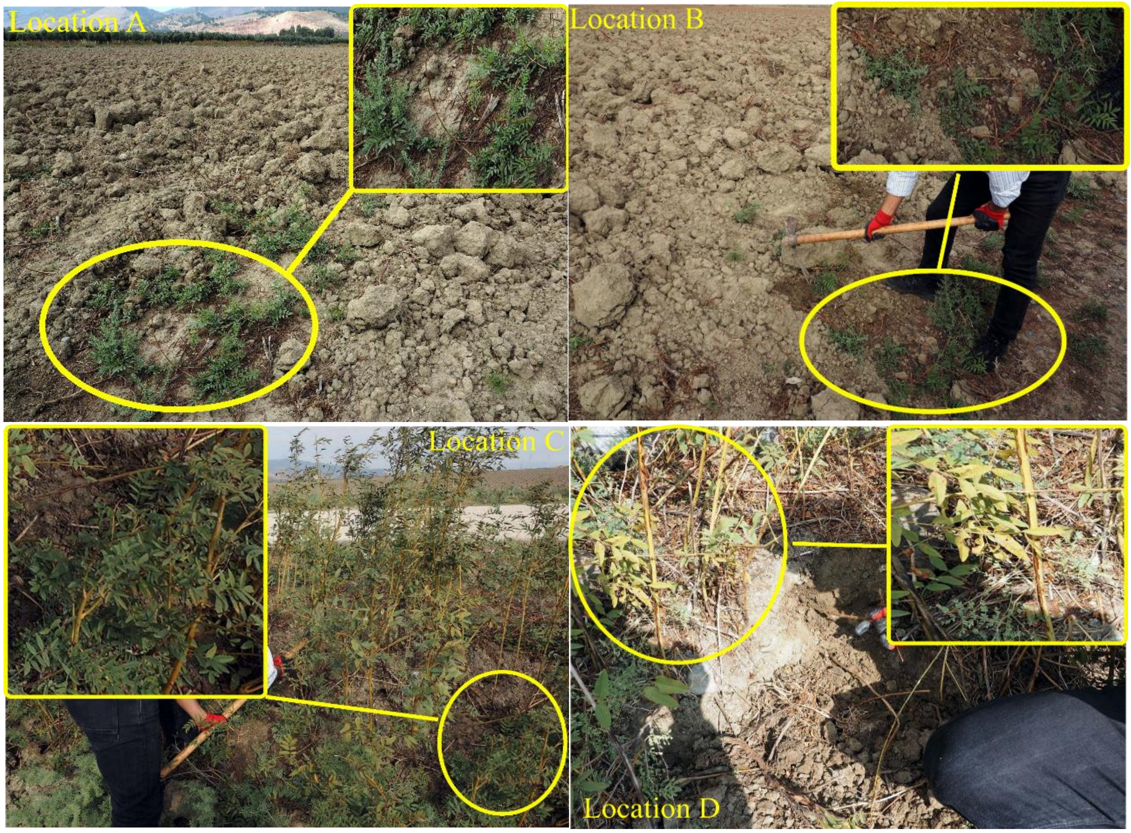

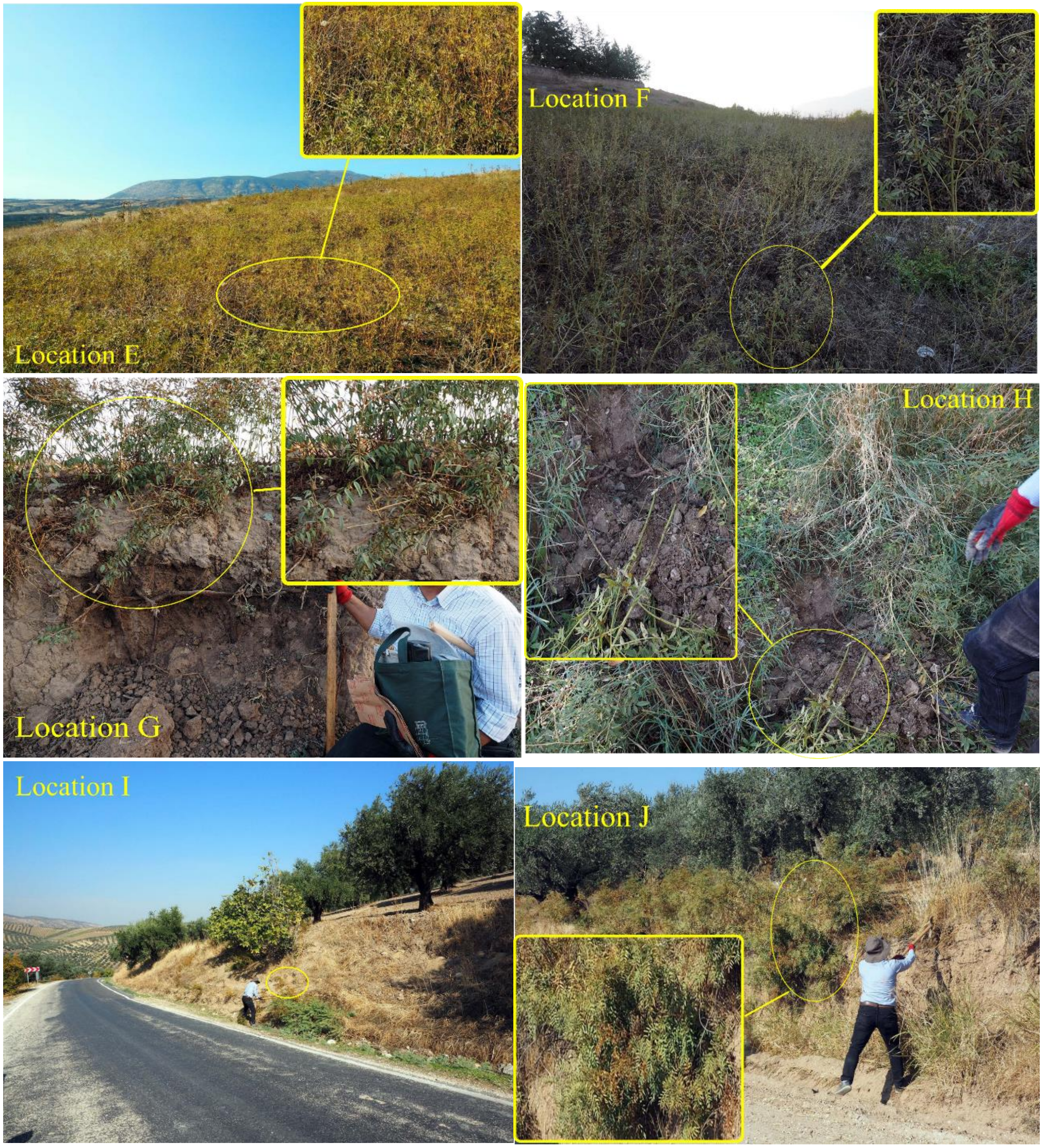
Location E
Location F
Location G
Location H
Location I
Location J
